# Gleanings from Our Exchanges

**Published:** 1872-01-01

**Authors:** 


### Gleanings from Our Exchanges.

### WHEN WILL SMALL-POX EPIDEMICS CEASE?

BY DR. E. MULLER (BERLIN),

*Geh. Med-Rath u. Director of the Imperial Vaccination Institute of Berlin.*

[From the Berlin Klin. Wochenschr.]

Epidemics of Small-Pox, often of malignant nature, are of no infrequent occurrence; and that, too, since the general introduction of vaccination. The immediate effect of such an epidemic upon the public is the increased tendency to vaccination. A subsequent effect is the seemingly strengthened claim of the opponents of vaccination of its lack of protective power, whereby vaccination is brought into disrepute. Highly opportune, then, is the question, why is it that, notwithstanding the fact that vaccination has been made compulsory, that but few individuals who have survived childhood remain unvaccinated, epidemics of small-pox unceasingly occur?

Investigations of this kind have long since led to the discovery of the fact that vaccination affords not a life-long, only a temporary prophylaxis. As a consequence, re-vaccination was adopted. This re-vaccination, however, has never been made a matter of legal compulsion except in the military service, where its adoption has furnished the most brilliant results. The authorities were content with merely recommending re-vaccination, and it must be confessed that this recommendation, in the rule, was but little heeded. It must also be confessed that if re-vaccination had been attempted to extent sufficient to stamp out a small-pox epidemic, the requisite amount of lymph would have been almost everywhere lacking. It would have been possible to obtain such a mass of lymph as whole communities would require, only by diluting it with glycerine. Thus the main hindrance to re-vaccination would have been overcome, and we may entertain the hope that every future epidemic may in this way soon be brought to an end. An indispensable condition to such a happy result is that the re-vaccination as well as the first vaccination shall have been properly performed.

As I have already shown in an essay "On Vaccination and the value of glycerine lymph for General Health" in Horn's Vierteljahrschr. 1869, Variola has become an unfashionable subject in the literature and among the faculties of Germany. Small-pox patients are hurried from the clinic as fast as possible. In the higher classes of society, where re-vaccination is seldomer neglected, small-pox occurs but rarely. The physician for the poor and for trades unions sends his variola patients as quickly as possible to the pest house. Hence human small-pox does not receive that consideration, that general medical interest, which is bestowed upon epidemics of other nature. Compare the scanty literature concerning the present small-pox epidemic with the abundance written every cholera season. The whole subject of vaccination, indeed, lies under comparative neglect. The only questions of interest seem to be the possibility of the transmission of syphilis, or the superiority of animal or human lymph. The majority of physicians are content to vaccinate from arm to arm with the virus they are able to command, and if called upon another day or in another place, they are not in supply of reliable virus from their own patients, but must beg it from the Institute or buy it at the drug store. No precaution is taken to lay by a supply for future use. Again, there is no little ignorance as to the proper method of its introduction, more carelessness as to the quality of the lymph employed. It is not of rare occurrence that individuals have been unsuccessfully re-vaccinated by their physicians, and afterward successfully re-vaccinated here at the Institute. According to my experience, the first re-vaccination almost always produces more or less genuine cow-pox pustules. When I hear physicians state, therefore, that they have vaccinated a large number of patients without any effect, I always consider myself justified in questioning the character of the lymph employed. Re-vaccinations of that kind react injuriously, not only upon the patients themselves by comforting them with the reflection that they are protected, but they are misfortunes for the whole subject of vaccination, as in case of a subsequent attack of small-pox the valuelessness of vaccination would seem to be demonstrated. Physicians, then, should never re-vaccinate with other lymph than that obtained from infants, and even then they should not be allowed to re-vaccinate unless they have obtained it themselves. Too often the physician fears public opinion to such extent that if he have no virus himself he will obtain at the drug store or elsewhere when he has no security whatever of its quality. Nor is it much better to re-vaccinate with animal lymph. As I have proven by my own experiments, a vaccination with animal lymph will not always succeed. Others confirm this in every particular. In the vaccination institute at Rotterdam where animal lymph is cultivated it is never used for re-vaccination on account of its uncertainty. When a re-vaccination with human lymph is repeated upon individuals unsuccessfully re-vaccinated with animal lymph, it will be seen that human lymph has the decided advantage. If then, an unsuccessful re-vaccination by animal lymph be ascribed to an existing protection of the individual, the same disrepute is cast upon vaccination as when the experiment is performed with defective human lymph. Epidemics of small-pox will cease, therefore, only when re-vaccination shall have become general, and not then unless the re-vaccinating physicians make use only of reliable lymph.—*The Clinic, Dec.* 23, 1871.

A Fully Matured Tænia Solium Expelled from a Child five days old.—The following case which occurred in the Long Island College Hospital, is reported by Samuel G. Armor, M.D., in the New York *Medical Journal*, December, 1871. A well developed and apparently healthy child on the fifth day after birth exhibited symptoms of intestinal irritation for which three $\frac{1}{6}$ gr. doses of calomel were ordered, to be followed by oil. Some ten hours after taking the first dose of calomel the infant passed, per anum, two segments of what was at once recognized to be tape worm. The specimen was carefully preserved and submitted to different members of the hospital staff, and was also placed under the microscope and the diagnosis concurred in that it was a well marked tænia solium. Additional segments of the worm were passed from time to time during the succeeding ten days, but none having the appearance of the head.

The writer comments on the case as follows: "The tænia solium according to Kuchmeister's investigations, only occurs in children who partake of hog's meat;" neither he nor Cobold makes mention of the possibility of a fully matured tænia occurring in infantile periods of life. The theory appears to have been generally accepted heretofore, that the encysted parasites are taken with the food into the stomach, and that the embryo set free from the covering of the egg by a process of digestion, passes into the intestine, fixes itself to the mucous membrane, and by a process of budding, produces the long, tape-like series of articulatious, which are finally converted into the full grown tænia. Whether this be the universally accepted theory, or not, certain it is that the encysted parasite, found in whatever part of the body, only develops to maturity in the intestinal canal. The query at once arises therefore, how did the cisticercus in the case here reported, gain entrance into the intestinal canal of the new born infant? for it is difficult to arrive at any other conclusion from the clinical history of the case, than that the worm was fully matured at the birth of the child.

Can the mother communicate the germs of the parasite to the fœtus in utero? and if so how do they gain entrance to the intestinal canal?

"To determine one of these questions, the mother, being still in the hospital, and having fully recovered from her confinement, was on the 8th of November—about two months after the birth of her child—put upon treatment for tapeworm; although neither previous history or present condition indicated the presence of tænia. After thoroughly evacuating the bowels, and while fasting she was ordered an emulsion of pumpkin seeds, which she faithfully took for twenty-four hours, at the end of which time she passed over seventy segments of tænia. This completes the clinical history of a case which throws much doubt upon the present received theories as to the probable and *exclusive* source of tænia. That the encysted parasites gain entrance to the stomach and bowels by means of animal food containing the parasytic germs, the experiments of Kuchmeister and others leave no room to doubt. But that they may gain entrance through the mother to the fœtus in utero would appear to be equally well established by this case."

Ovariotomy.—At a meeting of the Royal Med. and Chir. Soc., on the 13th of June, T. Spencer Wells presented a fourth series of one hundred cases of ovariotomy, which, following the order of former papers, he had arranged as follows:

Series 1. Cases in which ovariotomy was completed–100 cases: 78 recoveries, 22 deaths.

Series 2. Cases in which ovariotomy was commenced but not completed—6 cases: 2 relieved or cured, 4 died.

Series 3. Cases where an exploratory incision was made—7 cases: 5 recovered from incision, 2 died.

He showed that the mortality after ovariotomy was steadily diminishing. Of his first 100 cases, 34 died; of his second 100 cases, 28 died; of his third 100 cases, 23 died; and of his fourth 100, 22 died. Of this fourth series, 44 had been in hospital, and 56 in private practice. In private practice mortality was only 14 per cent., while in hospital it was 31 per cent. The author believed that the mortality in private practice might be taken as a guide to what might become the general average mortality after ovariotomy, and he was convinced that it might be reduced to about ten per cent. without excluding those extreme cases when the operation was performed as a last hope.—*Br. Med. Jour.*

Phosphates in Pregnancy.—Mr. Metcalfe Johnson, of Lancaster, recommends in the *Medical Times* the hydrated phosphate of lime of the British Pharmacopœia as a remedy for the sickness of pregnancy. He gives it in doses of from three to ten grains each, three times daily, suspended in water, and flavored according to the patient's taste. In some cases the relief has been so striking that patients have sent to ask for "some of that medicine that relieves the sickness." Mr. Johnson thinks the drug may supply phosphates to the nervous system, and also to the embryo, and that if phosphates be not supplied, the child may grow at the expense of the mother's osseous and nervous system.—*The Doctor.*

Bromide of Calcium.—The New York *Medical Journal* for December, contains a short note relative to this new preparation, by Wm. A. Hammond, M.D. He describes it as a white crystalline substance, very soluble in water, and readily decomposing on exposure to the atmosphere. Its taste is similar to that of bromide of potassium, though somewhat more pungent and disagreeable. The dose is from fifteen to thirty grains or more for an adult.

The action of bromide of calcium is similar to that of the other bromides, but it appears to be especially useful in those cases in which a speedy effect is desirable, as, owing to its stability, the bromide is readily set free and its peculiar action on the organism obtained more promptly than when either of the other bromides are administered. Chief among these effects is its hypnotic influence, and hence the bromide of calcium is particularly beneficial in case of delirium tremens, or in the insomania resulting from intense mental labor or excitement.

In those exhausted conditions of the nervous system attended with great irritability, such as are frequently met with in hysterical women, and a mental condition of extreme excitement, bromide of calcium has proved of decided service.

Diphtheria.—Dr. James E. Reeves, Wheeling, W. Va., (*Medical Times*) states that experience has abundantly satisfied him that the use of strong caustics is not advisable in the treatment of diphtheria—that in numerous instances they aggravate all the symptoms, and thus greatly endanger the patient. The tincture ferri chloridi may be applied in full strength to the diphtheritic patches and to the surface around their borders, by means of a camel's hair pencil; but even this practice he has generally abandoned during the last five or six years, and now contents himself with gargles or the atomized spray of the chlorine and iron mixture (℞ Potass. chlorat, ʒ ij.; acid hydrochloric puri, ʒ iss.; aquæ, ℥ vij.; tinc. ferri chloridi, ℥ j. M.) every hour or two when the patient is awake. At the same time this mixture may be internally administered, in the dose of from twenty drops to a teaspoonful, with or without a little simple syrup, every two or three hours, with the addition, if need be, of a little sulphate of quinia. Warm atomized inhalations of the chlorine and iron mixture, according to the above formula, promise, he thinks, the greatest hope in cases of diptheritic croup.—*Ibid.*

Treatment of Albuminoid Disease of the Kidney.—Dr. James H. Hutchinson, Physician to the Pennsylvania Hospital (*Medical Times*), says that the treatment of these diseases differs from that usually employed in other forms of Bright's disease. There is no indication to increase the flow of urine, for it is already sufficiently free, and the dropsy which is present depends simply upon the condition of the coats of the blood-vessels, which allows the ready passage of serum through them. The indication is rather to endeavor to prevent the loss of albumen, and with this view he prescribed to two of his patients ten grains of gallic acid four times daily, and for the older one, since he had a syphilitic history, ten grains of iodide of potassium, also three times daily. In the case of a boy, with the troublesome symptom, gastric irritability, he resorted to the use of lime-water and hydrocyanic acid and morphia. In many cases it resists every remedy, and, by the vomiting it induces, undoubtedly hastens the fatal issue, especially when diarrhœa exists at the same time — a coincidence which is by no means unfrequent in cases similar to these. These symptoms depend, he says, probably upon the disease of the blood-vessels of the stomach and intestines.—*Ibid.*

